# PA and OA induce abnormal glucose metabolism by inhibiting KLF15 in adipocytes

**DOI:** 10.1186/s12986-021-00628-2

**Published:** 2021-11-21

**Authors:** Cuizhe Wang, Xiaolong Chu, Yuchun Deng, Jingzhou Wang, Tongtong Qiu, Jiaojiao Zhu, Xin Yang, Chongge Pan, Jianyu Xiong, Jianxin Xie, Yongsheng Chang, Jun Zhang

**Affiliations:** 1 Medical College of Shihezi University, Bei-Er-Lu, Shihezi, 832000 Xinjiang China; 2 Department of Physiology and Pathophysiology, Tianjin Medical University, Tianjin, 300000 China

**Keywords:** Obesity, Palmitic acid, Oleic acid, Adipocyte, KLF15

## Abstract

**Background:**

Obesity-induced elevated serum free fatty acids (FFAs) levels result in the occurrence of type 2 diabetes mellitus (T2DM). However, the molecular mechanism remains largely enigmatic. This study was to explore the effect and mechanism of KLF15 on FFAs-induced abnormal glucose metabolism.

**Methods:**

Levels of TG, TC, HDL-C, LDL-C, and glucose were measured by different assay kits. qRT-PCR and Western Blot were used to detect the levels of GPR120, GPR40, phosphorylation of p38 MAPK, KLF15, and downstream factors.

**Results:**

KLF15 was decreased in visceral adipose tissue of obesity subjects and high-fat diet (HFD) mice. In HFD mice, GPR120 antagonist significantly promoted KLF15 protein expression level and phosphorylation of p38 MAPK, meanwhile reduced the blood glucose levels. While, blocking GPR40 inhibited the KLF15 expression. In 3T3-L1 adipocytes, 1500 μM PA inhibited KLF15 through a GPR120/P-p38 MAPK signal pathway, and 750 μM OA inhibited KLF15 mainly through GPR120 while not dependent on P-p38 MAPK, ultimately resulting in abnormal glucose metabolism. Unfortunately, GPR40 didn’t contribute to PA or OA-induced KLF15 reduction.

**Conclusions:**

Both PA and OA inhibit KLF15 expression through GPR120, leading to abnormal glucose metabolism in adipocytes. Notably, the inhibition of KLF15 expression by PA depends on phosphorylation of p38 MAPK.

**Supplementary Information:**

The online version contains supplementary material available at 10.1186/s12986-021-00628-2.

## Background

Nowadays, the incidence rate of obesity is gradually increasing in the world [1]. Epidemiological data show that obesity is an essential cause for insulin resistance (IR) and type 2 diabetes mellitus (T2DM) [1]. Most notably, elevated serum free fatty acids (FFAs) levels induced by obesity result in the occurrence of T2DM [2]. It has been widely acknowledged that FFAs can not only bring energy to the body, but also act as signal factors participating in numerous physiological activities depending on different carbon chain length [3]. FFAs can be divided into three categories, that is, short chain fatty acids (SCFAs) that are no more than 6 carbons, medium chain fatty acids (MCFAs) that are 6–12 carbons, and long chain fatty acids (LCFAs) that are more than 12 carbons [3]. Palmitic acid (PA, C16:0) and oleic acid (OA, C18:1) belonging to LCFAs are the main component of total FFAs and account for 27% and 31% respectively [4].

A large body of literature suggests that increased PA is a key risk factor for IR and T2DM [4–6]. A marked increase in synthesis of harmful complicated lipids, cell organelle dysfunction, and inflammatory reaction contribute to PA-induced IR and T2DM. Interestingly, the effects of OA on IR or T2DM are controversial. It has been reported that the OA emerges protective effects on insulin sensitivity [4]. While, these results are inconsistent with another study in which insulin sensitivity and glucose uptake were both abolished in 3T3-L1 adipocytes treated with PA and OA [7]. Similar results obtained Xie W et al*.* showed that decreased glucose transporter 4 (GLUT4) levels and impaired phosphorylation of serine/threonine protein kinase (AKT) were found in 3T3-L1 differentiated with OA, suggesting resistance to insulin in fat cells [8]. Often, G-protein-coupled receptor (GPR) 40 and GPR120 can act as nutrient sensors that are both activated by saturated or unsaturated LCFAs [9]. However, whether PA/OA-induced disordered glucose metabolism is GPR40/120 dependent is not very clear.

The Krüppel-like factors (KLFs) are vital regulators of cell development, differentiation, and activation [10]. KLF15 is expressed in numerous tissues, such as liver, adipose tissue, spleen, and skeletal muscle, and has significant effects in glucose and lipid metabolism in cells [10, 11]. It has been reported that KLF15 regulates the expression of genes related to glucose uptake and adipogenesis, such as *GLUT4* and *peroxisome proliferator activated receptor γ* (*PPARγ*) [10, 12]. Moreover, ADIPOLIN, an insulin-sensitizing and anti-inflammatory adipokine, could be transcriptionally activated by KLF15 in adipocytes [13]. Our results formerly demonstrated that compared with lean individuals, KLF15 mRNA expression levels of adipose tissue were markedly decreased in obese individuals, and had a negative correlation with body mass index (BMI), triglyceride (TG), low density lipoprotein cholesterol (LDL-C), while had a positive correlation with high density lipoprotein cholesterol (HDL-C), suggesting downregulated KLF15 expression may relevant to lipid metabolism triggered by obesity [14]. It has been reported that activation of P38 mitogen activated protein kinase (p38 MAPK) is integral to inhibit KLF15 expression in cardiac myocyte [15, 16]. Additionally, p38 MAPK is constantly regarded as signal downstream of Gq proteins that coupled with GPR120 or GPR40, suggesting Gq-p38 activation may be mediated by GPR120 or GPR40 [17]. However, the function of GPR120(40)/p38 MAPK/KLF15 in the development of FFA-related disordered glucose homeostasis remains ambiguous.

In the present study, we provide evidence that KLF15 was decreased in visceral adipose tissue of obesity subjects and high-fat diet (HFD) mice. Moreover, in HFD mice, we found that GPR120 antagonist significantly promoted KLF15 protein expression level, while blocking GPR40 inhibited the KLF15 expression. In 3T3-L1 adipocytes, KLF15 improved PA-induced abnormal glucose metabolism. Notably, PA inhibited KLF15 expression through GPR120/P-p38 MAPK signal pathway, and OA inhibits KLF15 expression mainly through GPR120 while not dependent on P-p38 MAPK, ultimately resulting in abnormal glucose metabolism of 3T3-L1 adipocytes.

## Methods

### Study subjects

60 patients with elective abdominal surgery in Xinjiang Kashgar people's Hospital from October to December 2012 and from April to September 2013 were collected. The 60 patients were separated into normal control (NC) group (18.5 kg/m^2^ ≤ BMI ≤ 24 kg/m^2^, n = 30) and obesity (OB) group (BMI ≥ 28 kg/m^2^, n = 30). General information of each subjects was collected and levels of fasting plasma glucose (FPG), HDL-C, LDL-C, TG, and total cholesterol (TC) were tested before surgery. Patients who underwent diabetes mellitus, tumors, acute inflammation, liver, and kidney disease, and took medicine in the period to treat diseases of glucose and lipid metabolism were excluded. The patients were all signed informed consent, and ethics was supplied by the Medical Ethics Committee at First Affiliated Hospital of Medical College of Shihezi University (Approval number: 2017–049-01).

### Experimental mouse models

36 four-week-old male C57BL/6 mice were purchased from Beijing Vital River Laboratory Animal Technology Co., Ltd. After acclimated for a week, they were randomly fed with normal diet including 10% fat ratio (ND, n = 8) and high fat diet including 60% fat ratio (HFD, n = 28) (Medicience Ltd, Jiangsu, China). The weight and body length of mice were recorded weekly. After 8 weeks of HFD feeding, 8 ND mice and 10 HFD mice were euthanized. The visceral adipose tissue and biochemical indicators were collected. The Lee’s index was calculated by [(body weight (g) × 10^3^)/body length (cm)]^1/3^. The other 18 HFD mice were start intraperitoneally injected with dimethylsulfoxide (DMSO, n = 6, Solarbio, Beijing, China), AH7614 (n = 6, 5 mg/kg, Tocris, Bristol, UK) or GW1100 (n = 6, 5 mg/kg, MCE, Monmouth, USA) every other day for four weeks. After the 18 HFD mice were put to execution, visceral tissue samples and biochemical indicators were collected. The experimental animal ethics were supplied by the Medical Ethics Committee at First Affiliated Hospital of Medical College of Shihezi University (Approval number: A2017-115-01).

### Cell lines and culture conditions

Mouse 3T3-L1 preadipocytes, purchased from the Chinese Academy of Sciences cell bank, were cultured in Dulbecco’s modified Eagle’s medium (DMEM, Gibco, California, USA) with 10% bovine calf serum (Biological Industries, Beth Haemek, Israel) and 1% penicillin at 37 °C under 5% CO_2_. After the 3T3-L1 preadipocytes were growing to the contact inhibition for 48 h, the culture medium was replaced with differentiation medium, which containing 0.5 mM isobutylmethylxanthine (IBMX, Sigma-Aldrich, St. Louis, USA), 5 µg/ml insulin (Sigma-Aldrich, St. Louis, USA), and 1 µM dexamethasone (Sigma-Aldrich, St. Louis, USA). 48 h later, the differentiation medium was replaced with DMEM supplemented 10% FBS and 5 µg/ml insulin. After another two days, the medium was replaced with DMEM containing 10% FBS for two more days. Lipid droplets in mature adipocytes were stained with Oil red O.

### Reagent preparation

40 mM PA (Sigma-Aldrich, St. Louis, USA) or OA (Sigma-Aldrich, St. Louis, USA) solution: PA (0.0307 g) or OA (30.08μL) were mixed to 3 mL of 0.1 mol/L sodium hydroxide (Fuyu Fine, Tianjin, China) solution, after that placing the mixture in a fully saponified water bath at 75℃ for about 30 min until the liquid becomes clear. Then, the mixture was immediately put into 3 mL of 40% bovine serum albumin solution without fatty acid (ZSGB-BIO, Beijing, China). 100 mmol/L AH7614 solution: 10 mg AH7614 was dissolved in 285 μL DMSO. 10 mmol/L GW1100 solution: 5 mg GW1100 was dissolved in 0.96 mL DMSO. 10 mmol/L SB203580 (MCE, Monmouth, USA) solution: 10 mg SB203580 was dissolved in 1.32 mL DMSO.

### Glucose consumption

3T3-L1 adipocytes were grown in six-well plates and induced differentiation into mature adipocytes. After PA or OA stimulation for 48 h, culture medium was collected for measurement of glucose concentration using the glucose oxidase method (cat# F006; Nanjing Jiancheng Biological Engineering Research Institute, Nanjing, China). The glucose consumption was calculated by the difference between glucose concentration for 0 h and glucose concentration for 48 h.

### Insulin sensitivity

After overexpression or silencing of KLF15 in mature adipocytes stimulated by PA for 48 h, the cell culture medium was discarded. Then, the cells were continuously stimulated with 1 ml insulin solution, which contained 100 nmol/L insulin, low glucose DMEM (1 g/L), and 10% FBS. The glucose concentration of medium was detected immediately, and determined as the glucose concentration of 0 min. The glucose concentration was then measured at 30, 60, 90, 120, 150, 180, 210, and 240 min. Finally, the glucose concentration curve was drawn.

### Quantitative reverse transcription PCR (qRT-PCR)

Total RNA was extract by Trizol (Thermo Fisher, New York, USA). In the reverse transcription (Eppendorf AG, Hamburg, Germany) system, 1 μg RNA was used to obtain 20 μL cDNA. The procedure set up with 42 °C for 60 min, afterwards 70 °C for 15 min. The mRNA expression levels of related genes were tested using the instrument Rotor-Gene Q (QIAGEN, Hilden, Germany). The procedure was as follows: 95 °C for 3 min, 45 cycles at 95 °C for 10 s, and 60 °C for 30 s. Glyceraldehyde 3-phosphate dehydrogenase (GAPDH) or β-actin was used as control for normalization. The record was represented by cycle threshold (CT) values, and the 2^−ΔCt^ or 2^−ΔΔCt^ was used for analysis. Additional file 1: Table S1 displays the primers of each gene.

### Western Blot

Cell lysates containing 1% phenylmethylsulfonyl fluoride (PMSF, Solarbio, Beijing, China) and radio-immunoprecipitation assay (RIPA) lysis buffer (Solarbio, Beijing, China) were used to lyse the cells and extract the total intracellular protein. The protein samples were mixed with 4 × loading buffer (Thermo Fisher, New York, USA) in a ratio of 3:1, and heated at 100℃ for 10 min. The gel-separated proteins were transferred onto the nitrocellulose (NC) filter membranes and then incubated with Tris buffered saline tween (TBST) buffer including 5% BSA for 2 h at ambient temperature. NC membranes were incubated at 4 °C overnight with antibodies to KLF15 (42 kDa; Abcam, 60B1220.1, 1:1000), GPR120 (42 kDa; Abcam, 60B1220.1, 1:1000), GPR40 (40 kDa; Thermofisher, D23G1, 1:1000), P-p38 MAPK (42 kDa; Cell Signaling, D1F2, 1:1000), GAPDH(36 kDa; ZSGB-BIO, TA-08, 1:1000), β-Tubulin (51 kDa; ZSGB-BIO, TA-09, 1:1000), β-actin (42 kDa; ZSGB-BIO, TA-10, 1:1000). The secondary antibody (ZSGB-BIO, 1:10,000) was incubated at ambient temperature for 2 h. The protein band results were identified by FluorChem HD2 (Thermo Scientific, Waltham, USA).

### Biochemical indicator test

Levels of TG, TC, HDL-C, LDL-C, and glucose were measured by TG assay kit (A110-1-1, Nanjing Jiancheng Bioengineering Institute, China), TC assay kit (A111-1-1, Nanjing Jiancheng Bioengineering Institute, China), HDL-C assay kit (A112-1-1, Nanjing Jiancheng Bioengineering Institute, China), LDL-C assay kit (A113-1-1, Nanjing Jiancheng Bioengineering Institute, China), and glucose assay Kit (F006-1-1, Nanjing Jiancheng Bioengineering Institute, China).

### Statistical analysis

Data are expressed as the mean ± SEM. Statistical significance was assessed using SPSS17.0 with the appropriate test: 2 tailed *t*-test or one-way ANOVA. A *P* < 0.05 was considered statistically significant.

## Results

### KLF15 is decreased in visceral adipose tissue of OB subjects

**Fig. 1:**
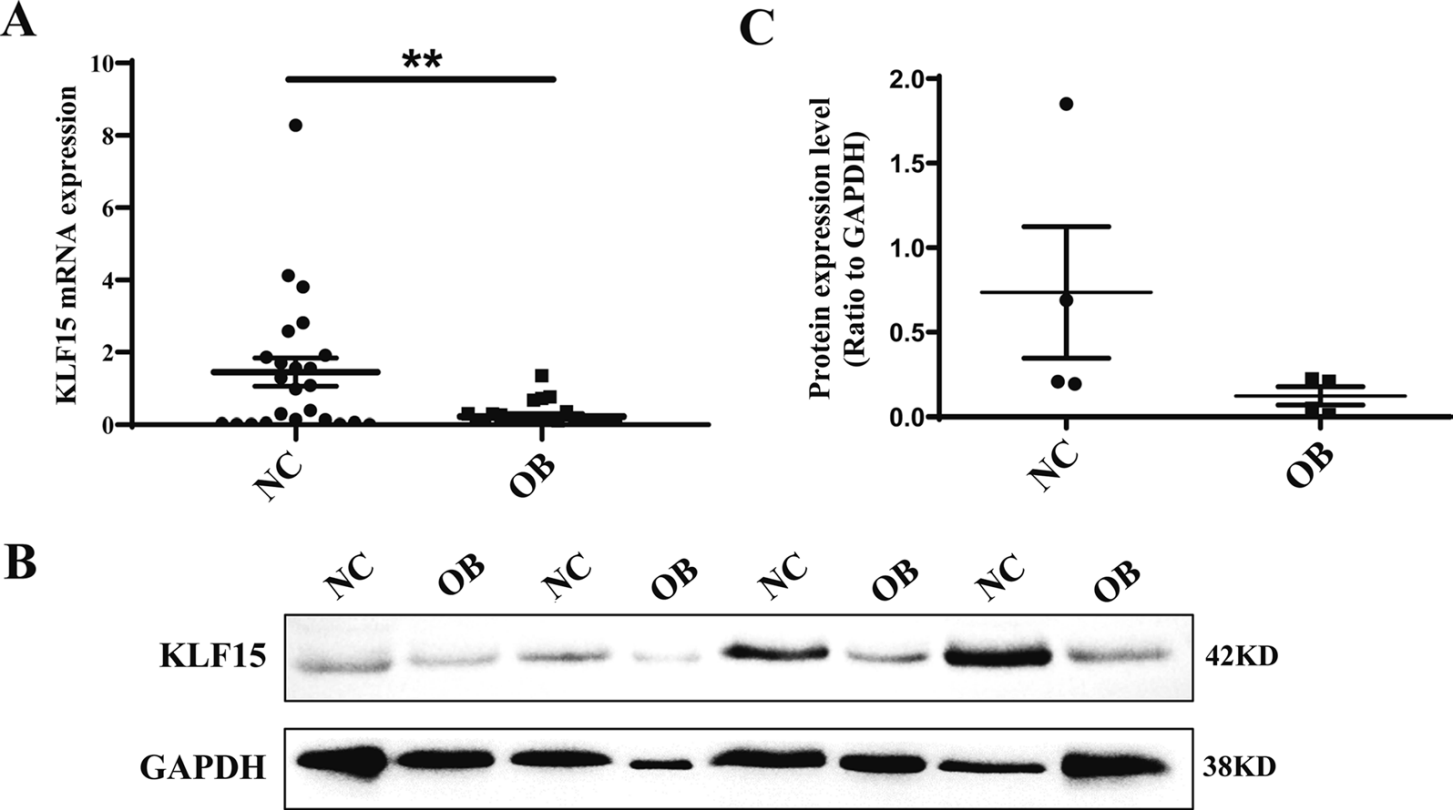
KLF15 mRNA and protein levels in visceral adipose tissue. mRNA expression levels (**A**) and protein expression levels **B** were detected. **C** Western Blot gray scale. Results represent mean ± SEM. *t* test, *P < 0.05 difference was statistically significant

General information and biochemical indexes of NC subjects (n = 30) and OB subjects (n = 30) were showed in Additional file 1: Table S2. We found that the weight, waist circumference (WC), Hip- circumference (HC), Waist-Hip Ratio (WHR), BMI, and TG were all significantly higher in OB group compared with NC group (*P* < 0.01), while the Age, FPG, HDL-C, LDL-C, and TC had no significant difference. The reduction was also observed for KLF15 mRNA and protein levels of visceral adipose tissue in OB subjects compared to NC subjects (Fig. 1A–C). KLF15 levels were then compared to general information and biochemical indexes to evaluate degree of correlation. The results showed that KLF15 levels were significantly and negatively correlated with WC, WHR, weight, BMI, and TG, suggesting KLF15 levels were correlated with obesity state (*P* < 0.05) (Additional file 1: Table S3).

### Reduced KLF15 expression of visceral adipose tissue is controlled by HFD

**Fig. 2:**
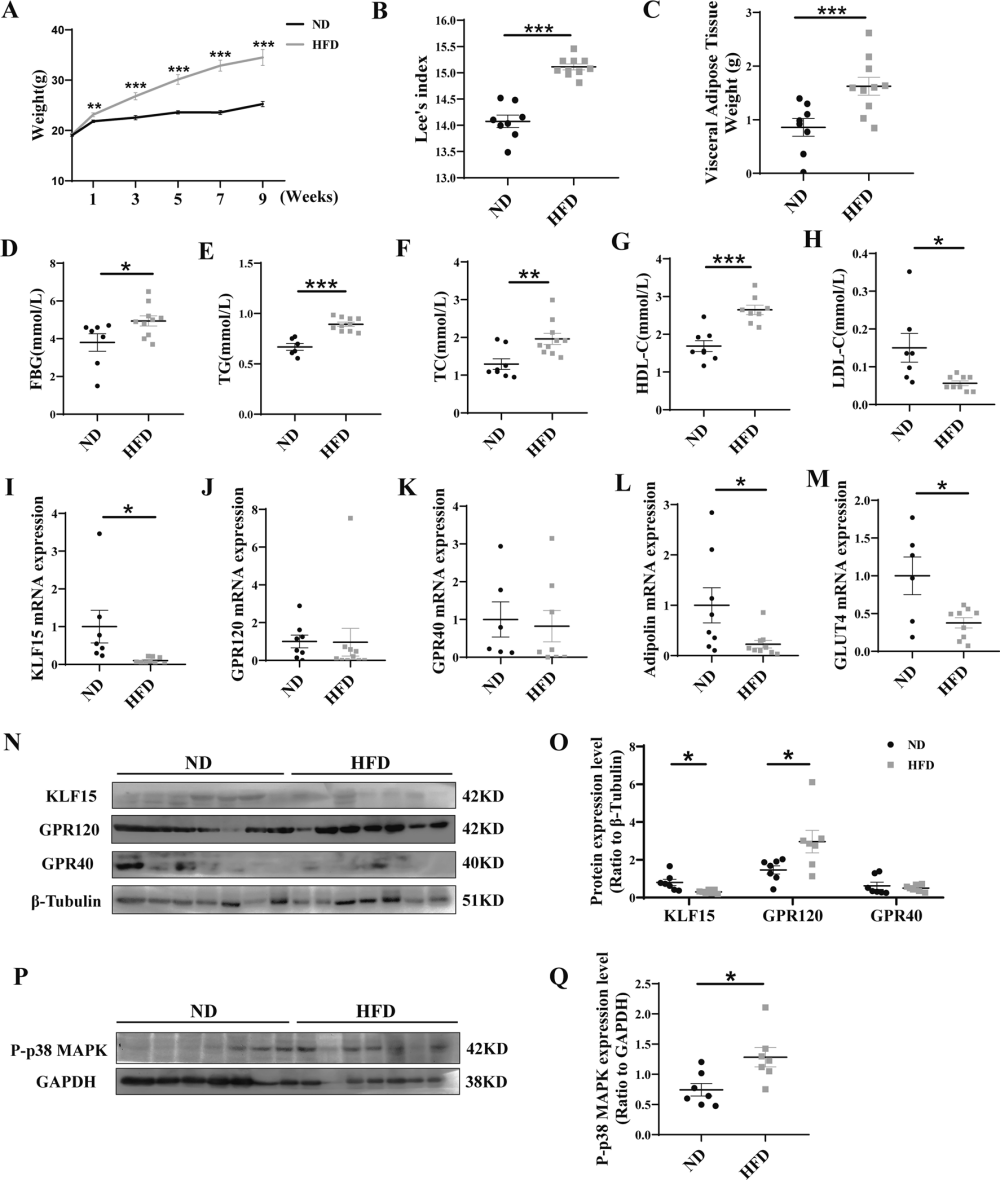
Mouse models of obesity was constructed and related genes expression were detected. Body weight **A** of ND and HFD mice was detected weekly. In the ninth week, Lee’s index (**B**) and visceral adipose tissue weight **C** were detected. Moreover, the levels of FBG (**D**), TG (**E**), TC (**F**), HDL-C (**G**), and LDL-C (**H**) were measured. In addition, the mRNA expression levels of related genes (**I**–**M**) and protein expression levels of related genes of visceral tissue (**N**, **P**) were tested. Western Blot gray scale (**O**, **Q**) were quantified. Results represent mean ± SEM. *t* test, *P < 0.05, **P < 0.01, ***P < 0.01 difference was statistically significant

As shown in Fig. 2A, the HFD-fed mice gained more weight than the ND-fed mice. In the ninth week, the Lee’s index and visceral adipose tissue weight were significantly higher in HFD group compared with ND group (*P* < 0.001) (Fig. 2B, C). In serum biochemical assays as shown in Fig. 2D–H, HFD group exhibited significant increase in serum fasting blood glucose (FBG), TG, TC, and HDL-C levels, and significant decrease in serum LDL-C level compared to ND groups (*P* < 0.05). Compared to the ND group, the KLF15, ADIPOLIN and GLUT4 mRNA expression levels of visceral adipose tissue were dramatically decreased in mice receiving HFD (*P* < 0.05) (Fig. 2I, L, M), while GPR120 and GPR40 mRNA expression had no significant difference (Fig. 2J, K). Moreover, in HFD group, the GPR120 protein expression level and the phosphorylation of p38 MAPK of visceral adipose tissue were significantly increased (*P* < 0.05), and the KLF15 protein expression level was significantly decreased (*P* < 0.05) (Fig. 2M–Q). More importantly, spearman correlation analysis revealed that KLF15 mRNA expression level was negatively relative with weight, Lee’s index, visceral adipose tissue weight, TG, TC, and HDL-C (*P* < 0.05), and significantly positive with ADIPOLIN and GLUT4 (*P* < 0.01) (Additional file 1: Table S4).

### KLF15 is upregulated by GPR120 antagonist and downregulated by GPR40 antagonist in HFD mice

**Fig. 3:**
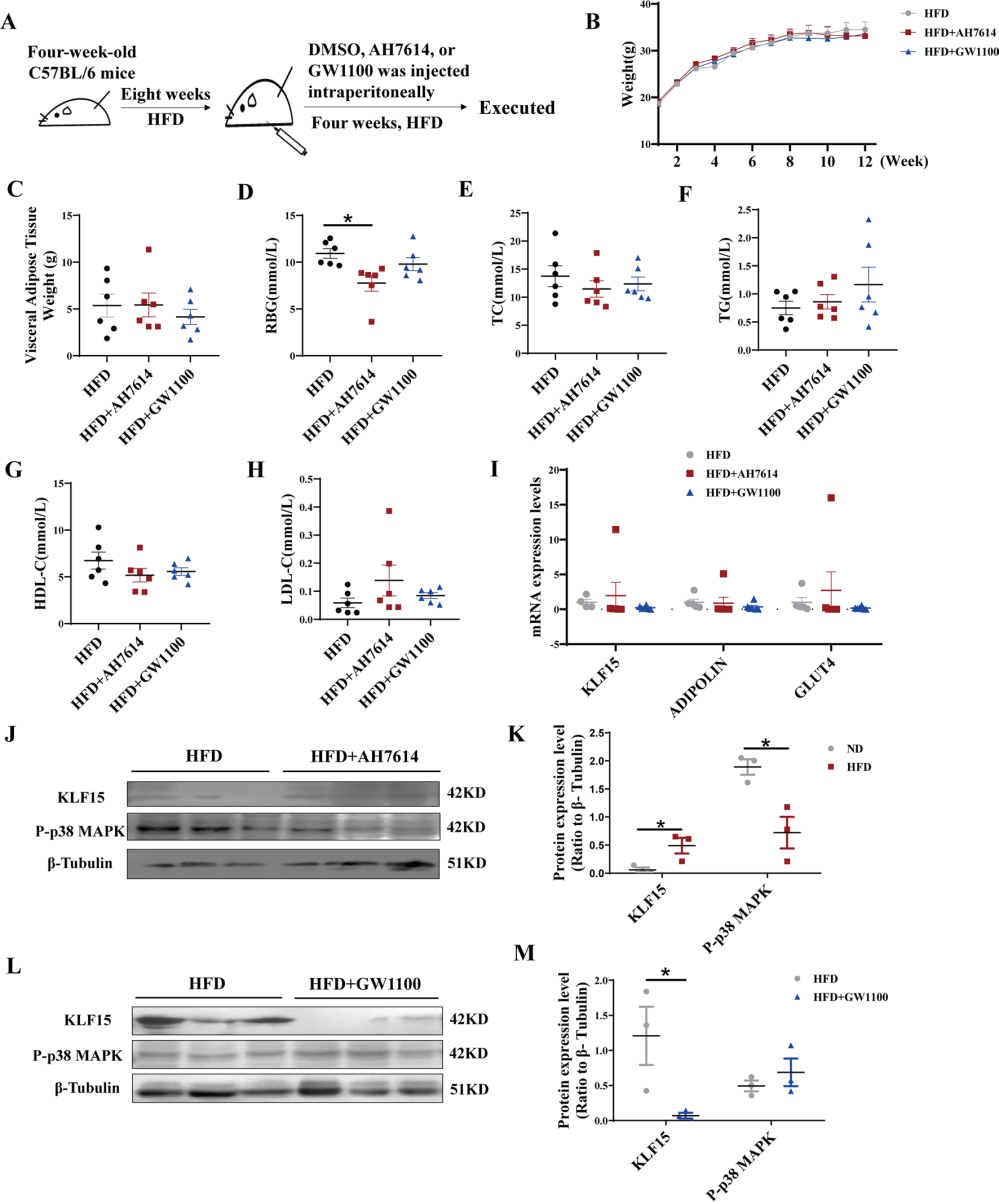
The effects of GPR120 and GPR40 antagonist in HFD mice. After HFD for eight weeks, the mice received an intraperitoneal injection of DMSO, GW1100 or AH7614 every other day for four weeks. The body weight **B** was detected in different weeks. In the twelfth week, the visceral adipose tissue weight (**C**), biochemical indexes (**D**–**H**), mRNA expression levels (**I**) and the protein expression levels (**J**, **L**) of related genes were detected. **K**, **M**)Western Blot gray scale. Results represent mean ± SEM. *t* test and One-way ANOVA, *P < 0.05 difference was statistically significant

To explore whether KLF15 expression was regulated by GPR40 or GPR120, the HFD mice received an intraperitoneal injection of GPR40 antagonist GW1100 or GPR120 antagonist AH7614 (Fig. 3A). As a result, GW1100 or AH7614 did not affect the whole body weight and visceral adipose tissue weight (Fig. 3B, C). The random blood glucose (RBG) levels in the HFD + AH7614 group were significantly decreased compared with the HFD group (*P* < 0.05) (Fig. 3D–H). The mRNA expression levels of KLF15, ADIPOLIN, GLUT4 in the HFD + AH7614 or HFD + GW1100 group were comparable to those observed in the HFD group (Fig. 3I). Of note, the KLF15 protein expression levels were significantly increased and the phosphorylation of p38 MAPK was significantly decreased in the HFD + AH7614 group (*P* < 0.05) (Fig. 3J, K). Conversely, the KLF15 protein expression levels were significantly decreased in the HFD + GW1100 group (*P* < 0.05), while the phosphorylation of p38 MAPK had no significant difference (Fig. 3L, M). Overall, these results exhibit that HFD-induced lower KLF15 expression of visceral adipose tissue is mainly regulated by GPR120.

### PA inhibits KLF15 expression through GPR120/P-p38 MAPK, resulting in abnormal glucose metabolism of 3T3-L1 adipocytes

**Fig. 4:**
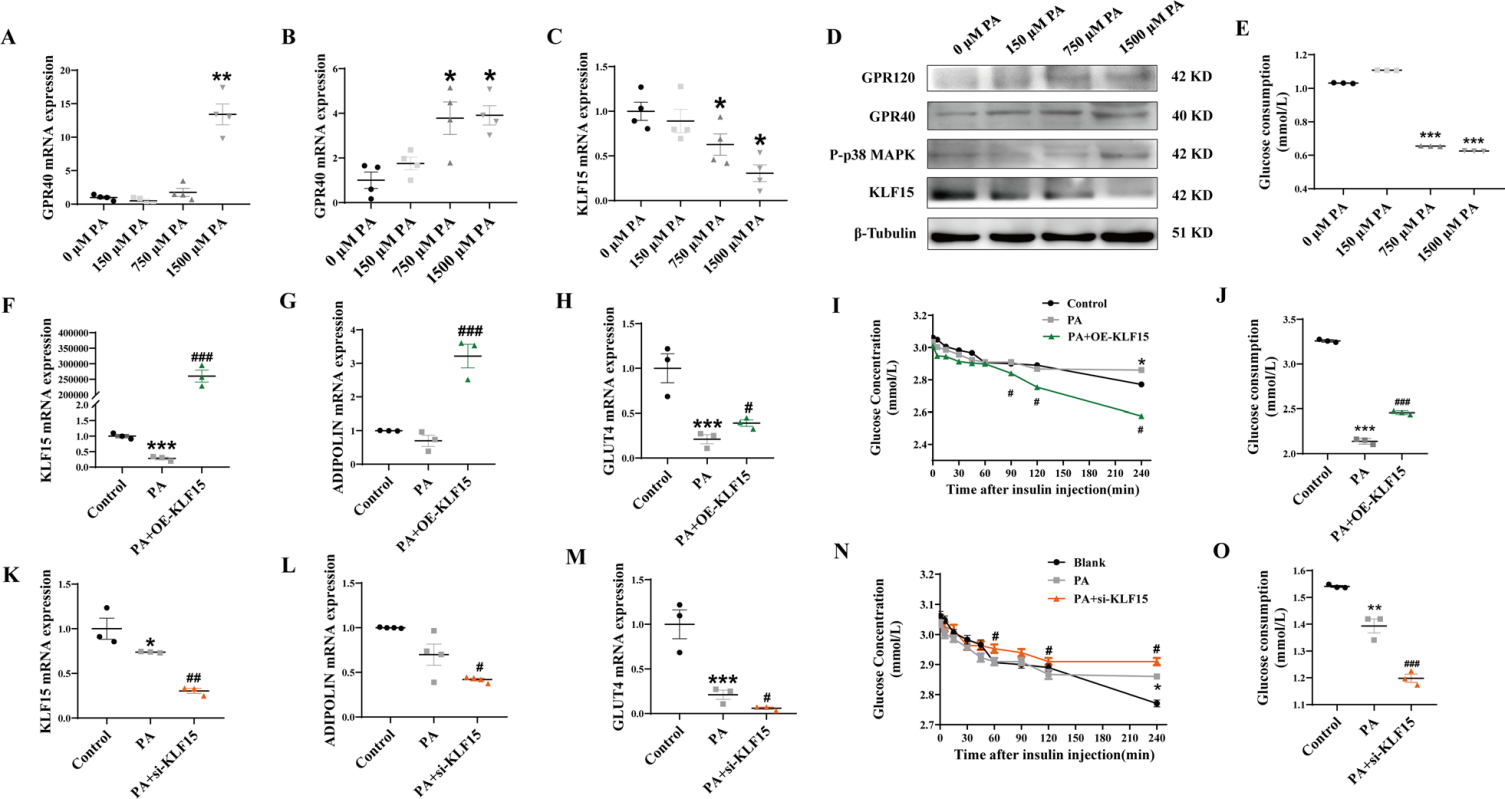
KLF15 improves PA-induced abnormal glucose metabolism in 3T3-L1 adipocytes. 3T3-L1 adipocytes were dealt with different concentrations of PA (0, 150, 750, and 1500 μM) for 48 h. The mRNA (**A**–**C**) and protein **D** expression levels of GPR120, GPR40, P-p38 MAPK, and KLF15 were detected; Glucose consumption **E** of 3T3-L1 adipocytes was tested. The expression of KLF15 was then overexpressed or silenced by transfection with overexpression plasmid (OE-KLF15) or interference fragment (si-KLF15). The mRNA **F**–**H**, **K**–**M** expression levels of KLF15, ADIPOLIN, GLUT4 and glucose consumption **J**, **O** were detected. After overexpression or silencing of KLF15 in mature adipocytes stimulated by PA for 48 h, the cells were continuously stimulated with 100 nmol/L insulin solution, and insulin sensitivity **I**, **N** were tested. Results represent mean ± SEM. One-way ANOVA, *P < 0.05, **P < 0.01 versus the control group, ^#^*P* < 0.05, ^##^*P* < 0.05, ^###^*P* < 0.001 versus the PA-treated group, difference was statistically significant

3T3-L1 adipocytes were dealt with different concentrations of PA to determine its effect on GPRs/P-p38 MAPK/KLF15 levels and glucose consumption. The results showed that compared with the 0 μM PA-treated group, the GPR40 and GPR120 mRNA expression levels were both significantly promoted and the KLF15 mRNA expression level was significantly reduced in 1500 μM PA-treated group (*P* < 0.05) (Fig. 4A–C). Western Blot results also discovered that 1500 μM PA markedly promoted the GPR40 and GPR120 expression and obviously inhibited the KLF15 expression. Also, in the presence of 1500 μM PA, p38-MAPK phosphorylation was increased (Fig. 4D). Moreover, the glucose consumption of 3T3-L1 adipocytes was obviously abrogated in 1500 μM PA-treated group (*P* < 0.001) (Fig. 4E).

Next, the expression of KLF15 in 3T3-L1 adipocytes was then overexpressed or silenced by transfection with overexpression plasmid (OE-KLF15) or interference fragment (si-KLF15) to investigate the role of KLF15 effects on PA-induced abnormal glucose metabolism. The results showed that KLF15 overexpression could rescue PA-mediated reduction of ADIPOLIN and GLUT4 expression (*P* < 0.05) (Fig. 4F–H). Moreover, KLF15 overexpression could significantly improve the effects of PA on impaired insulin sensitivity and glucose consumption (*P* < 0.05) (Fig. 4I, J). As expected, KLF15 silencing could significantly exacerbate the stimulatory effects of PA on reduced ADIPOLIN and GLUT4 expression, impaired insulin sensitivity and inhibited glucose consumption (*P* < 0.05) (Fig. 4K–O).

**Fig. 5:**
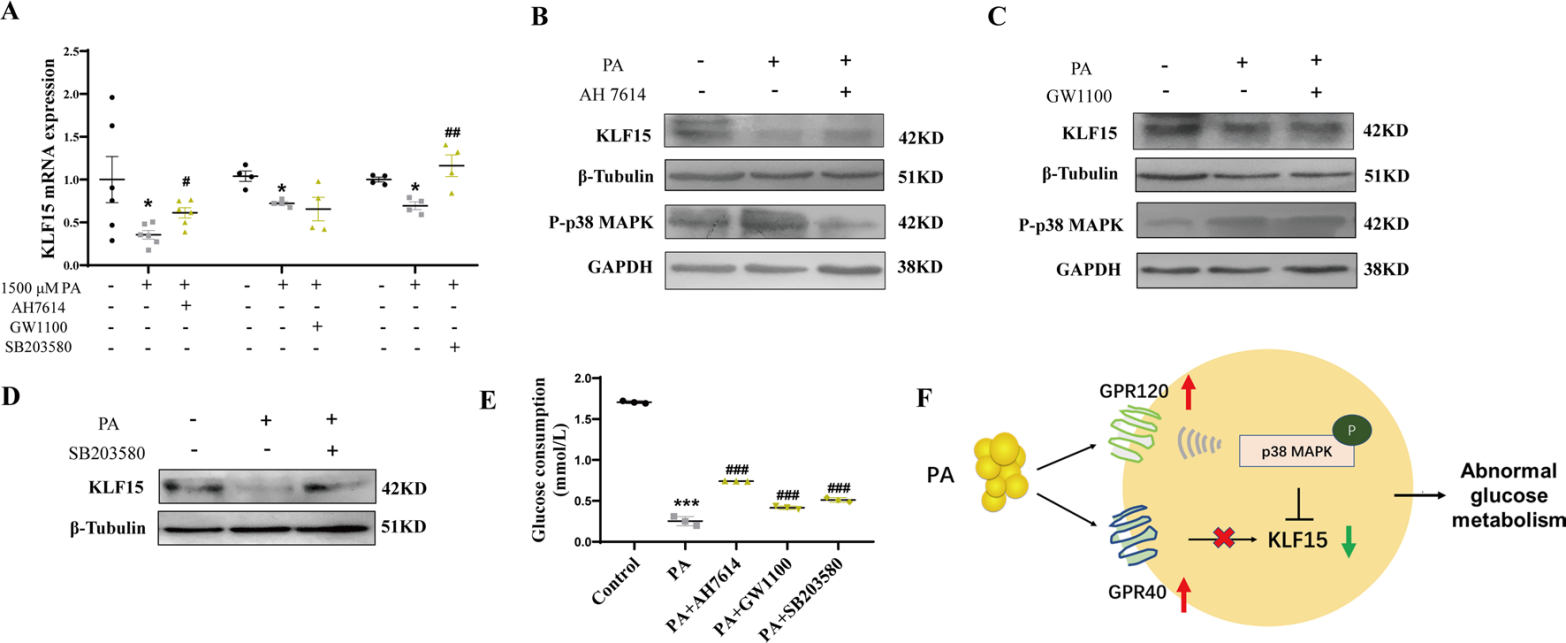
PA inhibits KLF15 expression through GPR120/P-p38 MAPK, resulting in abnormal glucose metabolism of 3T3-L1 adipocytes. AH7614, GW1100 and SB203580 were respectively added into 1500 μM PA treated cells to block GPR120, GPR40 and p38 MAPK. The mRNA (**A**) and protein **B**–**D** levels of P-p38 MAPK, KLF15 and glucose consumption **E** were detected. Pattern diagram **F** were presented. Results represent mean ± SEM. One-way ANOVA, *P < 0.05, ***P < 0.001 versus the control group, ^#^*P* < 0.05, ^##^*P* < 0.05, ^###^*P* < 0.001 versus the PA-treated group, difference was statistically significant

To further investigate whether PA inhibited KLF15 expression and glucose consumption through a GPRs/P-p38 MAPK pathway, AH7614, GW1100 and SB203580 were respectively added into 1500 μM PA treated 3T3-L1 adipocytes to block GPR120, GPR40 and phosphorylated p38 MAPK. We found that PA-induced suppressed levels of KLF15 and elevated P-p38 MAPK, were overturned in 3T3-L1 adipocytes when cotreated with AH7614 (Fig. 5A, B). While, the levels of P-p38 MAPK and KLF15 were not affected by GW1100 (Fig. 5A, C). Moreover, KLF15 expression levels were obviously increased when blocking phosphorylated p38 MAPK (*P* < 0.05) (Fig. 5A, D). Meanwhile, the glucose consumption was partly but significantly improved in PA cotreated with AH7614, GW1100 and SB203580 (*P* < 0.05) (Fig. 5E). The above results suggest that PA inhibits KLF15 expression through GPR120/P-p38 MAPK, resulting in abnormal glucose metabolism of 3T3-L1 adipocytes (Fig. 5F).

### OA inhibits KLF15 expression mainly through GPR120 while not dependent on P-p38 MAPK, resulting in abnormal glucose metabolism of 3T3-L1 adipocytes

**Fig. 6:**
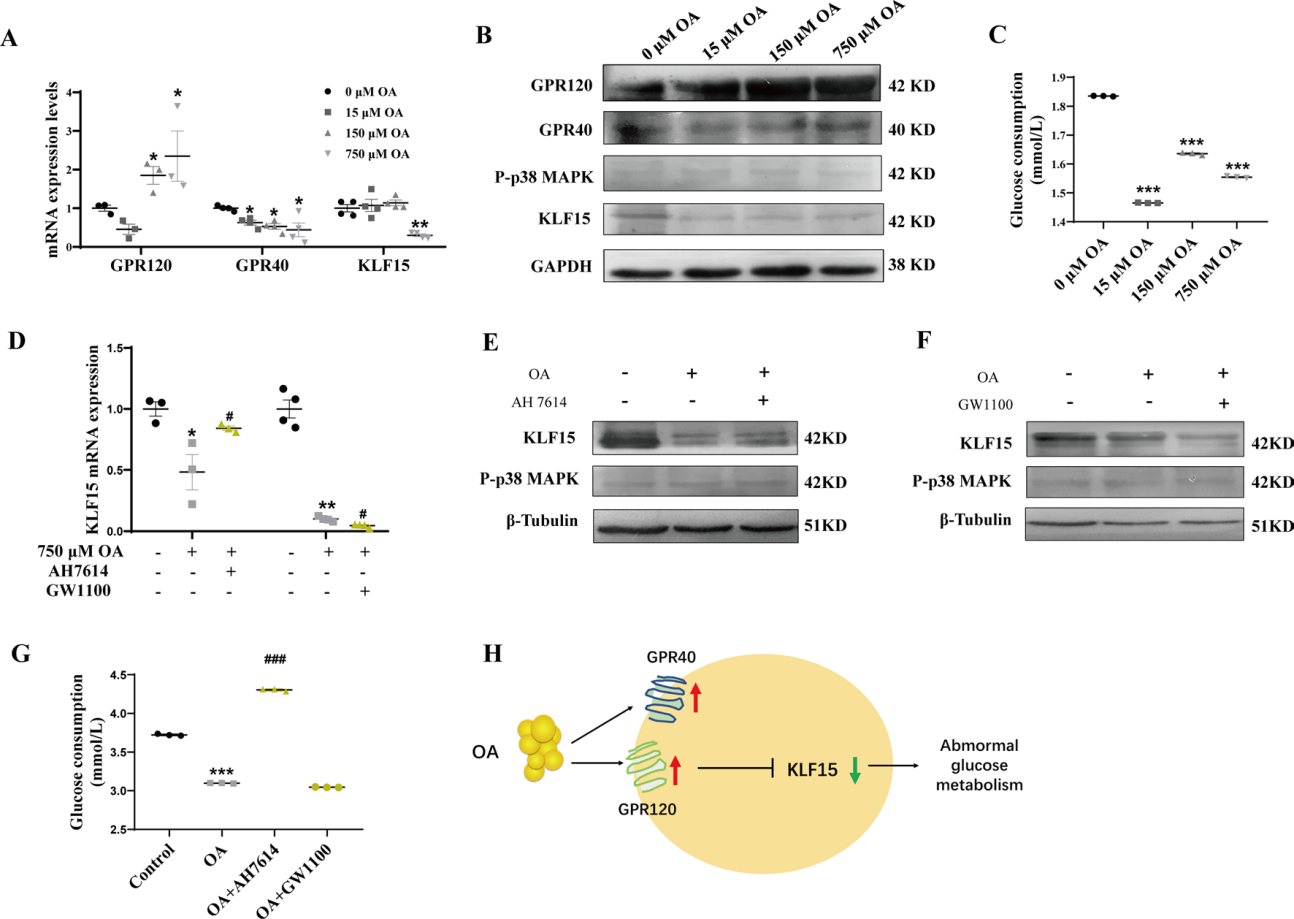
OA inhibits KLF15 expression mainly through GPR120 while not dependent on P-p38 MAPK, resulting in abnormal glucose metabolism of 3T3-L1 adipocytes. 3T3-L1 adipocytes were treated with different concentration OA (0, 15, 150, and 750 μM) for 48 h. The mRNA (**A**) and protein **B** expression levels of GPR120, GPR40, P-p38 MAPK, and KLF15 were detected; Glucose consumption **C** of 3T3-L1 adipocytes was tested. AH7614 and GW1100 were respectively added into 1500 μM PA treated cells to block GPR120 and GPR40. The mRNA (**D**) and protein **E**–**G** levels of P-p38 MAPK, KLF15 and glucose consumption **H** were detected. Pattern diagram I were presented. Results represent mean ± SEM. One-way ANOVA, *P < 0.05, **P < 0.01 versus the control group, ^#^*P* < 0.05, ^###^*P* < 0.001 versus the OA-treated group, difference was statistically significant

3T3-L1 adipocytes were dealt with different concentrations of OA to determine its effect on GPRs/P-p38 MAPK/KLF15 pathway and glucose consumption. The results indicated that compared with the 0 μM OA-treated group, the GPR120 mRNA and protein expression levels were significantly increased in 750 μM OA-treated group (*P* < 0.05) (Fig. 6A, B). Moreover, in 750 μM OA-treated group, although GPR40 mRNA expression level was slightly but significantly decreased (*P* < 0.05), GPR40 protein expression level was slightly increased (Fig. 6A, B). In addition, KLF15 mRNA and protein expression levels were both obviously abrogated in 750 μM OA-treated group (*P* < 0.05) (Fig. 6A, B). While, the p38-MAPK phosphorylation had no significant difference (Fig. 6B), which suggested that OA had no effect on p38-MAPK phosphorylation. Moreover, the glucose consumption of 3T3-L1 adipocytes was also obviously abrogated in 750 μM OA -treated group (*P* < 0.001) (Fig. 6C).

To further investigate whether OA inhibited KLF15 expression and glucose consumption through GPR120 or GPR40, AH7614 and GW1100 were respectively added into 750 μM OA treated 3T3-L1 adipocytes to block GPR120 and GPR40. We found that OA-induced suppressed KLF15 levels and glucose consumption were overturned in 3T3-L1 adipocytes when cotreated with AH7614 (*P* < 0.05) (Fig. 6D, E, G). In contrast to cotreated with AH7614, OA-induced suppressed KLF15 levels were exacerbated when cotreated with GW1100 (*P* < 0.05) (Fig. 6D, F), which indicated that GPR40 may partly promoted KLF15 expression. But unfortunately, the glucose consumption didn’t alter in OA cotreated with GW1100 group compared with OA-treated group (Fig. 6G). Whatever in OA cotreated with AH7614 or GW1100 group, p38-MAPK phosphorylation had no significantly difference. These results suggest that OA inhibits KLF15 expression mainly through GPR120 while not dependent on P-p38 MAPK, resulting in abnormal glucose metabolism of 3T3-L1 adipocytes (Fig. 6H).

## Discussion

T2DM is associated with increased total FFAs concentrations, which induced by obesity [18]. While, the specific mechanisms responsible for these effects of FFAs are not well understood. It has been reported that PA and OA are the main component of total FFAs and account for 27% and 31% respectively [4]. Previous studies indicate that PA-induced GPR40 activation weakens insulin sensitivity in N43/5 hypothalamic neuronal cells [19]. By contrast, PA-stimulated GPR40-deficient beta cells exhibited less insulin secretion [20]. In adipocytes from morbidly obese subjects, tumor necrosis factor α (TNF-α) and Interleukin-6 (IL-6) expression were significantly increased under PA stimulation, while the effects were reversed by silencing GPR120 [21]. Moreover, OA can act as GPR120 agonists and an OA-abundant diet ameliorated whole-body IR in obese mice by inhibiting inflammatory reaction of adipose tissue, liver, and skeletal muscle, reducing macrophage infiltration, and promoting IL-10 expression level, while all these influences were blocked by GPR120 silencing [22, 23]. In addition, OA increases insulin sensitivity through a GPR40-calcium signaling pathway in HepG2 cells [24]. These findings indicate that both PA and OA can activate GPR40 and GPR120. More importantly, it's not hard to speculate that PA or OA may have a different behavior in response to different GPRs. Meanwhile, GPR40 or GPR120 may play different biological functions due to different FFAs.

Consistently, our data demonstrated that both PA and OA can upregulate GRP40 and GPR120 expression. Moreover, our findings also showed that both PA and OA caused glucose consumption reduction of 3T3-L1 adipocytes through GPR120, which was in contradiction with previous research indicating that a GPR120-selective activator could improve IR and chronic inflammation in HFD mice [25, 26]. One possible reason is that GPR120 is usually the functional receptor for ω-3 FFAs, which can exhibit potent effects on resisting inflammation and improving insulin sensitivity [25, 26]. While, when its ligand is PA or OA, the different effects may be produced. Additionally, our data also showed that PA-mediated glucose consumption reduction of 3T3-L1 adipocytes also relied on GPR40. While, GPR40 antagonist didn’t influence the OA-induced glucose consumption reduction although OA promoted the GPR40 expression, suggesting OA inhibited glucose consumption not dependent on GPR40. In large part, the reason could be that OA had slight promoting effects on GPR40, which is expressed in adult β cells of rodents with little constitutive expression in adipose tissue [20]. Therefore, the effect on phenotype was not obvious although we blocked GPR40. Besides, it has been reported that the effect of FFAs on promoting insulin secretion had positive correlation with chain length and saturation level [27]. Thus, the saturation degree of PA and OA may lead to different physiological effects, while further experiment remains to be elucidated.

In this study, we also assessed the molecular mechanism of PA/OA-mediated abnormal glucose metabolism. Here, we showed that KLF15 is decreased in omental adipose tissue of obesity subjects. More importantly, KLF15 levels were significantly and negatively correlated with WC, WHR, Weight, BMI, and TG, suggesting KLF15 levels were correlated with obesity state. Moreover, it has been reported that KLF15 can regulate glucose uptake, and activation of p38 MAPK is essential to reduce KLF15 expression [15, 16]. Thus, it is logical to speculate that high levels of FFAs, particularly PA or OA, may regulate glucose metabolism through P-p38 MAPK/KLF15 in adipose tissue. Obviously, this study demonstrated that KLF15 protein expression was substantially increased and the phosphorylation of p38 MAPK was significantly decreased after blocking GPR120 in obesity animal model. Moreover, PA-induced abnormal glucose metabolism through a GPR120/P-p38 MAPK/KLF15 signal pathway in 3T3-L1 adipocytes, while GPR40 had no effects on P-p38 MAPK under PA stimulation. Interestingly but inconsistently, it has been reported that Gq-p38 activation was mediated by GPR40 rather than GPR120 involving in autophagy of breast cancer cells [28]. Probably, the activation mechanism of p38 MAPK may be distinct in different cell type or pattern.

Unfortunately, OA-induced abnormal glucose metabolism through GPR120/KLF15 signal pathway in 3T3-L1 adipocytes, while not depend on P-p38 MAPK. This may be associated with other important factors that can also mediate KLF15 effects on glucose consumption when OA stimulate 3T3-L1 adipocyte, such as inflammatory factors TNF-α that reduced the expression of KLF15 in chondrocytes and vascular endothelial cells [29, 30]. Moreover, whatever PA or OA stimulation, GPR40 didn’t contribute to the elevated phosphorylation of p38 MAPK and reduced KLF15 expression. In sharp contrast, blocking GPR40 inhibited the KLF15 expression in *vivo* and *vitro*. In other words, GPR40 may promoted KLF15 expression in 3T3-L1 adipocytes, while the possible mechanism needs to be further investigated. Overall, these results indicated that PA inhibited KLF15 expression through GPR120/P-p38 MAPK signal pathway, and OA inhibits KLF15 expression mainly through GPR120 while not dependent on P-p38 MAPK, ultimately resulting in abnormal glucose metabolism of 3T3-L1 adipocytes.

## Conclusions

This work identifies PA and OA promotes abnormal glucose metabolism by inhibiting KLF15 expression in 3T3-L1 adipocytes.

## Supplementary Information

**Additional file1**. **Table S1.** Primers sequence used in real-time PCR. **Table S2.** Comparison of general information, biochemical indexes between NC and OB subjects. **Table S3.** Correlation of KLF15 mRNA expression with general information and biochemical indexes in subjects. **Table S4.** Correlation of KLF15 mRNA expression with general information and biochemical indexes in mice.

## Data Availability

The data and material used to support the findings of this study are available from the corresponding author upon request.

## Abbreviations

IR: Insulin resistance
T2DM: Type 2 diabetes mellitus
FFAs: Free fatty acids
SCFAs: Short chain fatty acids
MCFAs: Medium chain fatty acids
LCFAs: Long chain fatty acids
PA: Palmitic acid
OA: Oleic acid
GLUT4: Glucose transporter 4
AKT: Serine/threonine protein kinase
GPR: G-protein-coupled receptor
KLFs: Krüppel-like factors
PPARγ: Peroxisome proliferator activated receptor γ
BMI: Body mass index
TG: Triglyceride
LDL-C: Low density lipoprotein cholesterol
HDL-C: High density lipoprotein cholesterol
p38 MAPK: P38 mitogen activated protein kinase
FPG: Fasting plasma glucose
TC: Total cholesterol
DMSO: Dimethylsulfoxide
DMEM: Dulbecco’s modified Eagle’s medium
IBMX: Isobutylmethylxanthine
PMSF: Phenylmethylsulfonyl fluoride
RIPA: Radio-immunoprecipitation assay
TBST: Tris buffered saline tween
TNF-α: Tumor necrosis factor α
IL-6: Interleukin-6

## References

[1] Wueest S, Konrad D (2018). The role of adipocyte-specific IL-6-type cytokine signaling in FFA and leptin release. Adipocyte.

[2] Arner P, Rydén M (2015). Fatty acids, obesity and insulin resistance. Obes Facts.

[3] Miyamoto J, Hasegawa S, Kasubuchi M, Ichimura A, Nakajima A, Kimura I (2016). Nutritional signaling via free fatty acid receptors. Int J Mol Sci.

[4] Palomer X, Pizarro-Delgado J, Barroso E, Vázquez-Carrera M (2018). Palmitic and oleic acid: the yin and yang of fatty acids in type 2 diabetes mellitus. Trends Endocrinol Metab.

[5] Muscarà C, Molonia MS, Speciale A, Bashllari R, Cimino F, Occhiuto C, Saija A, Cristani M (2019). Anthocyanins ameliorate palmitate-induced inflammation and insulin resistance in 3T3-L1 adipocytes. Phytother Res.

[6] Zhang X, Wang Y, Ge HY, Gu YJ, Cao FF, Yang CX, Uzan G, Peng B, Zhang DH (2018). Celastrol reverses palmitic acid (PA)-caused TLR4-MD2 activation-dependent insulin resistance via disrupting MD2-related cellular binding to PA. J Cell Physiol.

[7] Malodobra-Mazur M, Cierzniak A, Dobosz T (2019). Oleic acid influences the adipogenesis of 3T3-L1 cells via DNA Methylation and may predispose to obesity and obesity-related disorders. Lipids Health Dis.

[8] Xie W, Hamilton JA, Kirkland JL, Corkey BE, Guo W (2006). Oleate-induced formation of fat cells with impaired insulin sensitivity. Lipids.

[9] Suckow AT, Polidori D, Yan W, Chon S, Ma JY, Leonard J, Briscoe CP (2014). Alteration of the glucagon axis in GPR120 (FFAR4) knockout mice: a role for GPR120 in glucagon secretion. J Biol Chem.

[10] Gray S, Feinberg MW, Hull S, Kuo CT, Watanabe M, Sen-Banerjee S, DePina A, Haspel R, Jain MK (2002). The Krüppel-like factor KLF15 regulates the insulin-sensitive glucose transporter GLUT4. J Biol Chem.

[11] Du X, Rosenfield RL, Qin K (2009). KLF15 Is a transcriptional regulator of the human 17beta-hydroxysteroid dehydrogenase type 5 gene. A potential link between regulation of testosterone production and fat stores in women. J Clin Endocrinol Metab.

[12] Gray S, Wang B, Orihuela Y, Hong EG, Fisch S, Haldar S, Cline GW, Kim JK, Peroni OD, Kahn BB, Jain MK (2007). Regulation of gluconeogenesis by Krüppel-like factor 15. Cell Metab.

[13] Enomoto T, Ohashi K, Shibata R, Kambara T, Uemura Y, Yuasa D, Kataoka Y, Miyabe M, Matsuo K, Joki Y, Hayakawa S, Hiramatsu-Ito M, Ito M, Murohara T, Ouchi N (2013). Transcriptional regulation of an insulin-sensitizing adipokine adipolin/CTRP12 in adipocytes by Krüppel-like factor 15. PLoS ONE.

[14] Wang C, Ha X, Li W, Xu P, Gu Y, Wang T, Wang Y, Xie J, Zhang J (2016). Correlation of A2bAR and KLF4/KLF15 with obesity-dyslipidemia induced inflammation in uygur population. Mediators Inflamm.

[15] Leenders JJ, Wijnen WJ, Hiller M, van der Made I, Lentink V, van Leeuwen RE, Herias V, Pokharel S, Heymans S, de Windt LJ, Høydal MA, Pinto YM, Creemers EE (2010). Regulation of cardiac gene expression by KLF15, a repressor of myocardin activity. J Biol Chem.

[16] Hoa N, Ge L, Korach KS, Levin ER (2018). Estrogen receptor beta maintains expression of KLF15 to prevent cardiac myocyte hypertrophy in female rodents. Mol Cell Endocrinol.

[17] Slone S, Anthony SR, Wu X, Benoit JB, Aube J, Xu L, Tranter M (2016). Activation of HuR downstream of p38 MAPK promotes cardiomyocyte hypertrophy. Cell Signal.

[18] Sobczak IS, Blindauer A, Stewart J (2019). Changes in plasma free fatty acids associated with type-2 diabetes. Nutrients.

[19] Hernández-Cáceres MP, Toledo-Valenzuela L, Díaz-Castro F, Ávalos Y, Burgos P, Narro C, Peña-Oyarzun D, Espinoza-Caicedo J, Cifuentes-Araneda F, Navarro-Aguad F, Riquelme C, Troncoso R, Criollo A, Morselli E (2019). Palmitic acid reduces the autophagic flux and insulin sensitivity through the activation of the free fatty acid receptor 1 (FFAR1) in the hypothalamic neuronal cell line N43/5. Front Endocrinol (Lausanne).

[20] Steneberg P, Rubins N, Bartoov-Shifman R, Walker MD, Edlund H (2005). The FFA receptor GPR40 links hyperinsulinemia, hepatic steatosis, and impaired glucose homeostasis in mouse. Cell Metab.

[21] Rodriguez-Pacheco F, Gutierrez-Repiso C, Garcia-Serrano S, Alaminos-Castillo MA, Ho-Plagaro A, Valdes S, Garcia-Arnes J, Gonzalo M, Andrade RJ, Moreno-Ruiz FJ, Rodriguez-Cañete A, Martinez-Ferriz A, Garcia-Fuentes E (2017). The pro-/anti-inflammatory effects of different fatty acids on visceral adipocytes are partially mediated by GPR120. Eur J Nutr.

[22] Sawamura R, Kawabata Y, Kawabata F, Nishimura S, Tabata S (2015). The role of G-protein-coupled receptor 120 in fatty acids sensing in chicken oral tissues. Biochem Biophys Res Commun.

[23] Oliveira V, Marinho R, Vitorino D, Santos GA, Moraes JC, Dragano N, Sartori-Cintra A, Pereira L, Catharino RR, da Silva AS, Ropelle ER, Pauli JR, De Souza CT, Velloso LA, Cintra DE (2015). Diets containing α-linolenic (ω3) or oleic (ω9) fatty acids rescues obese mice from insulin resistance. Endocrinology.

[24] Wu HT, Chen W, Cheng KC, Ku PM, Yeh CH, Cheng JT (2012). Oleic acid activates peroxisome proliferator-activated receptor δ to compensate insulin resistance in steatotic cells. J Nutr Biochem.

[25] Oh DY, Walenta E, Akiyama TE, Lagakos WS, Lackey D, Pessentheiner AR, Sasik R, Hah N, Chi TJ, Cox JM, Powels MA, Di Salvo J, Sinz C, Watkins SM, Armando AM, Chung H, Evans RM, Quehenberger O, McNelis J, Bogner-Strauss JG, Olefsky JM (2014). A Gpr120-selective agonist improves insulin resistance and chronic inflammation in obese mice. Nat Med.

[26] Oh DY, Talukdar S, Bae EJ, Imamura T, Morinaga H, Fan W, Li P, Lu WJ, Watkins SM, Olefsky JM (2010). GPR120 is an omega-3 fatty acid receptor mediating potent anti-inflammatory and insulin-sensitizing effects. Cell.

[27] Briscoe CP, Tadayyon M, Andrews JL, Benson WG, Chambers JK, Eilert MM, Ellis C, Elshourbagy NA, Goetz AS, Minnick DT, Murdock PR, Sauls HR, Shabon U, Spinage LD, Strum JC, Szekeres PG, Tan KB, Way JM, Ignar DM, Wilson S, Muir AI (2003). The orphan G protein-coupled receptor GPR40 is activated by medium and long chain fatty acids. J Biol Chem.

[28] Zhu S, Lin G, Song C, Wu Y, Feng N, Chen W, He Z, Chen YQ (2017). RA and ω-3 PUFA co-treatment activates autophagy in cancer cells. Oncotarget.

[29] Li Y, Zhao M, Xiao W (2018). KLF15 regulates the expression of MMP-3 in human chondrocytes. J Interferon Cytokine Res.

[30] Liu B, Xu L, Yu X, Li W, Sun X, Xiao S, Guo M, Wang H (2018). Protective effect of KLF15 on vascular endothelial dysfunction induced by TNF-α. Mol Med Rep.

