# Supplementary material for: PA and OA induce abnormal glucose metabolism by inhibiting KLF15 in adipocytes
Source: Nutr Metab (Lond). 2021 Nov 21;18:100. doi: 10.1186/s12986-021-00628-2 (PMC8607635; doi:10.1186/s12986-021-00628-2)
Supplement: Supplementary file 1 — Additional file1. Table S1. Primers sequence used in real-time PCR. Table S2. Comparison of general information, biochemical indexes between NC and OB subjects. Table S3. Correlation of KLF15 mRNA expression with general information and biochemical indexes in subjects. Table S4. Correlation of KLF15 mRNA expression with general information and biochemical indexes in mice. [file 12986_2021_628_MOESM1_ESM.docx]

**Table S1 Primers** **sequence used in real-time PCR**

| **Primer name** | **Primer (5'-3')** | **Fragment(bp)** | |
| --- | --- | --- | --- |
| *Human-KLF15–F* | GGTACGGCTTCACACCTGAG | | 150 |
| *Human-KLF15–R* | ACACCAAAAGCAGCCACCT | |  |
| *Human- GAPDH-F* | GGTGGTCTCCTCTGACTTCAA | | 211 |
| *Human-GAPDH-R* | TCTTCCTCTTGTGCTCTTGCT | |  |
| *Mouse-KLF15–F* | GCGAGAAGCCCTTTGCCT | | 99 |
| *Mouse- KLF15–R* | GCTTCACACCCGAGTGAGAT | |  |
| *Mouse-GPR40–F* | AGAGGCTTACGCTGAGCTTG | | 196 |
| *Mouse-GPR40–R* | AAGAAAAGGGATGGCCAGAT | |  |
| *Mouse-GPR120–F* | TTTCATAAACCCGGACCTAGGA | | 90 |
| *Mouse-GPR120–R* | CCAGTGACCAGTGGGTTGAGT | |  |
| *Mouse-Adipolin–F* | TACCACGTCCAACCGTGAG | | 84 |
| *Mouse-Adipolin–R* | GTCATGTGGGCATCTGAGAG | |  |
| *Mouse-GLUT-4–F* | GGTGTGGTCAATACGGTCTTCAC | | 128 |
| *Mouse-GLUT-4–R* | AGCAGAGCCACGGTCATCAAGA | |  |
| *Mouse-β-actin–F* | CGTTGACATCCGTAAAGACC | | 281 |
| *Mouse-β-actin–R* | AACAGTCCGCCTAGAAGCAC | |  |

**Table S2** **Comparison of general information,** **biochemical indexes between NC and OB subjects**

| **Testing index** | **NC** | **OB** |
| --- | --- | --- |
| Case number | 30 | 30 |
| Age | 48.66±18.77 | 47.93±10.90 |
| Weight (kg) | 61.40±8.20 | 82.67±9.26*** |
| WC (cm) | 87.10±13.90 | 115.19±7.77*** |
| HC (cm) | 92.62±7.93 | 109.67±9.27*** |
| WHR | 0.93±0.12 | 1.06±0.09*** |
| BMI | 22.08±2.34 | 31.95±3.59*** |
| FPG (mmol/L) | 5.06±0.86 | 5.37±0.76 |
| HDL-C (mmol/L) | 1.37±0.68 | 1.63±0.95 |
| LDL-C (mmol/L) | 3.09±1.033 | 2.95±0.98 |
| TG (mmol/L) | 2.85±1.91 | 4.17±2.04** |
| TC (mmol/L) | 5.52±0.95 | 4.93±1.21 |

Results represent mean ± SEM. *t* test，***P<*0.01，****P<*0.001 difference was statistically significant.

**Table S3 Correlation of KLF15 mRNA expression with general information**

**and biochemical indexes in subjects**

| **Index** | **R** | ***p*-value** | **n** |
| --- | --- | --- | --- |
| WC | -0.433** | 0.002 | 47 |
| HC | -0.278 | 0.058 | 47 |
| WHR | -0.325* | 0.026 | 45 |
| FPG | -0.024 | 0.871 | 47 |
| weight | -0.362* | 0.012 | 47 |
| BMI | -0.362** | 0.012 | 47 |
| HDL-C | 0.012 | 0.943 | 38 |
| LDL-C | -0.143 | 0.392 | 38 |
| TG | -0.343* | 0.035 | 38 |
| TC | 0.069 | 0.711 | 31 |

Pearson correlation analysis, ******P*<0.05, *******P*<0.01 the correlation was statistically significant.

**Table S4 Correlation of KLF15 mRNA expression with general information**

**and biochemical indexes in mice**

| **Index** | **R** | ***p*-value** | **n** |
| --- | --- | --- | --- |
| Weight | -0.720* | 0.02 | 16 |
| Lee’s Index | -0.714** | 0.002 | 16 |
| Visceral adipose tissue weight | -0.565* | 0.023 | 16 |
| FBG | -0.393 | 0.147 | 15 |
| TG | -0.718** | 0.004 | 14 |
| TC | -0.696** | 0.003 | 16 |
| HDL-C | -0.550* | 0.034 | 15 |
| LDL-C | 0.457 | 0.100 | 14 |
| GPR120 | 0.388 | 0.137 | 16 |
| GPR40 | 0.154 | 0.633 | 12 |
| ADIPOLIN | 0.868*** | 0.000 | 16 |
| GLUT4 | 0.569** | 0.034 | 14 |

Spearman correlation analysis, **P*<0.05, ***P*<0.01, ****P*<0.001 the correlation was statistically significant.
